# Surface Quality Enhancement of Fused Deposition Modeling (FDM) Printed Samples Based on the Selection of Critical Printing Parameters

**DOI:** 10.3390/ma11081382

**Published:** 2018-08-08

**Authors:** Mercedes Pérez, Gustavo Medina-Sánchez, Alberto García-Collado, Munish Gupta, Diego Carou

**Affiliations:** 1 Department of Mechanical and Mining Engineering, University of Jaén, EPS de Jaén, Campus LasLagunillas, 23071 Jaén, Spain; mpj00011@red.ujaen.es (M.P.); gmedina@ujaen.es (G.M.-S.); acollado@ujaen.es (A.G.-C.); 2 Mechanical Engineering Department, National Institute of Technology, Hamirpur, H.P. 177005, India; munishguptanit@gmail.com; 3 Centre for Mechanical Technology and Automation (TEMA), University of Aveiro, Campus Santiago, 3810-193 Aveiro, Portugal

**Keywords:** 3D printing, additive manufacturing, ANOVA, correlation coefficients, fused deposition modeling, non-parametric tests, surface roughness

## Abstract

The present paper shows an experimental study on additive manufacturing for obtaining samples of polylactic acid (PLA). The process used for manufacturing these samples was fused deposition modeling (FDM). Little attention to the surface quality obtained in additive manufacturing processes has been paid by the research community. So, this paper aims at filling this gap. The goal of the study is the recognition of critical factors in FDM processes for reducing surface roughness. Two different types of experiments were carried out to analyze five printing parameters. The results were analyzed by means of Analysis of Variance, graphical methods, and non-parametric tests using Spearman’s *ρ* and Kendall’s τ correlation coefficients. The results showed how layer height and wall thickness are the most important factors for controlling surface roughness, while printing path, printing speed, and temperature showed no clear influence on surface roughness.

## 1. Introduction

### 1.1. Additive Manufacturing

Currently, the manufacturing industry is a sector highly globalized with a constant need for productivity gains and innovation. In this regard, additive manufacturing (AM) is considered to be one of the latest manufacturing revolutions and a future leading edge technology [1]. Additive manufacturing is entering the market to meet the demand of custom parts of complex geometry and reduce investment in tooling. Nowadays, this manufacturing process is still considered as a promising technology and is studied extensively in order to assess its viability in commercial applications such as electronics (resistors and sensors), optical (antennas), medical (artificial hip joints, bone structures, and tissue scaffolds), automotive, communication, and aerospace industries (engines, turbines, and thermal insulation coatings) [2]. Despite the great improvements that have been made in recent years, additive manufacturing still has some limitations. For instance, Oropalloand Piegl [3] identified ten challenges that should be conveniently studied and solved in coming years, such as shape optimization, design for 3D printing, or pre- and post-processing.

Additive manufacturing is characterized by the manufacture of pieces from a CAD model through the accumulation and joining of layers for obtaining the desired physical model. Recently, ASTM International defined a body of terms for additive manufacturing [4]. The different types of processes can be classified depending on [5]: (a) raw material (liquid, powder or solid); and (b) the kind of physical joint between the material. Currently, there are available several processes such as Stereolithography (SLA) [6], Selective Laser Sintering/Melting (SLS/SLM) [7], Laminated Object Manufacturing (LOM) [8], and Fused Deposition Modeling (FDM) [9,10].

The selection of the additive manufacturing process must take into account the pros and cons of each of the technologies. For instance, the FDM process is simple, which makes it a suitable candidate for being chosen by general users. Its main advantages are [10,11,12,13]: low machinery cost, no expensive tooling is necessary, broad range of materials, high durability of the components, acceptable dimensional accuracy, and not being time consuming. But, the process also has some disadvantages, such as low mechanical strength, difficulty to obtain thin walls, and poor surface quality. Polymers are widely used as the main base material in FDM processes. Typical materials include PLA [14] and ABS [15], but composite materials are also being adopted for manufacturing complex components. For instance, Fe-nylon6 composite wires were compared to ABS solutions, concluding that the composite materials are highly wear resistant [16].Polypropylene reinforced with glass-fiber wasstudied, showing adequate mechanical properties for small series of parts [17].

Although FDM processes have significant industrial value for manufacturing complex components, there is a need tocarry out proper research focused on prominent aspects such as surface roughness and performance optimization. The performance of the manufactured parts depends upon a large number of process factors, such as the type of material and process parameters, so it is quite difficult to obtain an ideal FDM process that fulfils all the requirements, particularly producing products of high surface quality.

### 1.2. Surface Quality in FDM Processes

In additive manufacturing, in general, pre-processing and post-processing activities should be carried out [3]. However, the quality of the parts is not adequate when compared to other mature manufacturing processes, such as machining. One of the main problems for obtaining good surface quality in additive manufacturing is the staircase effect. According to Strano et al. [18], usually manual post-processing operations are needed for obtaining adequate surface roughness because complex geometries compromise the advantages of additive manufacturing. Pandey et al. [19] analyzed the staircase effect that generates “chordal error” between an original surface of a CAD model and the corresponding triangle in the tessellated model. The authors concluded that the tessellation and slicing during the manufacturing process are two sources of surface inaccuracies that must be taken into account.

Various studies have been specifically carried out on FDM process parameters, discussing their effect on outputs, such as mechanical properties, and surface topography and quality [20,21,22]. For instance, Altan et al. [14] studied the effect of process parameters on surface roughness and the tensile strength on polylactic acid (PLA) samples. The samples were fabricated as per the ASTM standards and a Taguchi L16 experimental design, using three parameters: layer thickness, deposition head velocity, and nozzle temperature. The authors concluded that the layer thickness and deposition head velocity are dominant factors on surface roughness.

Campbell et al. [23] investigated surface roughness for different materials. The authors found that, in the case of ABS material, when using layer thickness of 0.253 mm, the surface roughness values for FDM processes ranged between 9 μm and 40 μm. Recently, Akande et al. [24] analyzed the optimal process parameters for obtaining good surface finish and dimensional accuracy. The authors employed a layer height of 0.25 and 0.5 mm, varying the filling density and speed of deposition, identifying that the surface roughness for PLA material ranged between 2.46 μm and 22.48 μm. Altan et al. [14] used layer thickness between 0.1 mm and 0.4 mm to create PLA samples using a FDM process. The surface roughness obtained varied within the range of 9.102 to 10.275 μm.

From previous scientific records, it has been identified that the performance of the FDM process extensively depends upon its process parameters and their levels. However, the number of publications dealing with the identification of critical factors and the optimization of the manufacturing process depending on adequate selection of factors and levels is still limited, particularly when it comes to surface roughness.

The optimization of the process parameters used for printing is an adequate strategy for improving part performance in terms of surface finish. So, the present paper addresses the study of surface roughness for FDM pieces, which has not been studied in detail in the literature. The paper shows an experimental study on fused deposition modeling, analyzing the quality of the parts after varying a set of printing parameters: layer height, wall thickness, printing speed, temperature, and printing path using both statistical and graphical methods.

## 2. Materials and Methods

### 2.1. Materials

**Figure 1:**
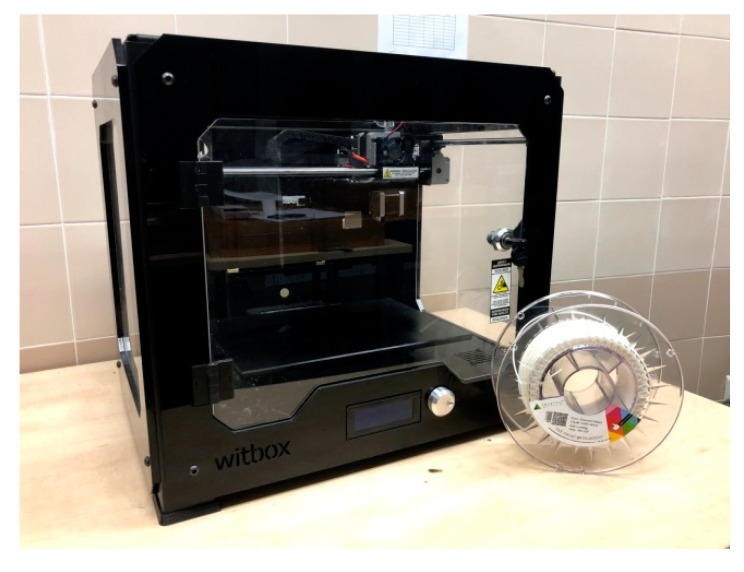
WITBOX printer and PLA filament.

The 3D printer used was a WITBOX printer (Figure 1) by BQ manufacturer (Madrid, Spain) using FDM technology. It is equipped with 0.4 mm diameter nozzle and glass cold base, A4-size (297 × 210 mm). The recommended printing speed is 50 mm/s and the maximum is 80 mm/s. The own-design extruder has a blower for cooling printed objects. The software used and recommended by the WITBOX printer manufacturer is Ultimaker Cura Software 3.2.1., which allows using STL and G-Code standards.

Polylactic acid (PLA) (Smart Materials 3D, Alcalá la Real, Spain) has been used as base material (Table 1). The company that provided the material was Smart Materials 3D. It does not incorporate recycled or recovered material. It is fully stabilized and it has a diameter of 1.75 mm with a variability of ±0.03 mm in diameter. Besides, no warping is expected.

**Figure 2:**
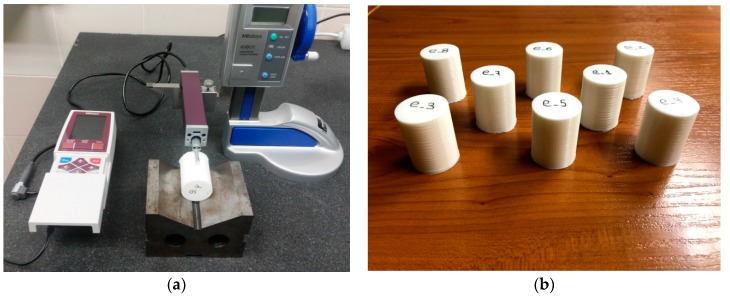
(**a**) Surface roughness measurement setup (**b**) Printed samples for experiment 1.

Surface roughness was measured using a handheld Mitutoyo Surftest SJ-210 (Mitutoyo, Kawasaki, Japan) profilometer (Figure 2a) with sampling length of 2.5 mm and measuring speed of 0.5 mm/s. With this technology, cylindrical samples such as those shown in Figure 2b were measured.

### 2.2. Experimental Plan

In additive manufacturing, there are several factors that could influence surface roughness, such as [25,26,27,28]:

- Material extrusion: temperature, viscosity, density, type of material, and mechanical properties.
- Chamber: temperature, pressure, vibrations, position of the platform, position of the extruder, system coordinates, and heat evacuation.
- Extruder: speed, angle of inclination, diameter of extrusion, vibration, and acceleration.
- Deposition characteristics: building direction, wall thickness, layer height, orientation, external geometry, and speed.

Due to the high number of factors, a selection of factors to carry out a more economical and practical study was made. The experimental investigation was divided into two stages. The first stage was designed as a screening stage [29] to identify the most critical printing factors for surface roughness. The second stage was performed in order to increase the knowledge of the printing factors based on the results of the first stage.

All printed samples had dimensions of 30 mm in diameter and 40 mm in height. The first analysis was done to study the influence of layer height, wall thickness, printing speed, and temperature (material). These factors were varied using two levels: minimum and maximum. So, eight tests were performed by means of a fractional factorial design of four factors with two levels. Fractional factorial designs allow carrying out experimental studies with limited number of experiments and, thus, reducing cost and time.

For layer height, values of 0.15 and 0.25 mm were chosen. The first one is the minimum recommended by the predefined options of the Cura software. The second one is a higher value, which was selected expecting an increase in surface roughness as it was identified in the literature. For printing speed, a value of 40 mm/s was selected; a speed lower than that recommended by the printer manufacturer, and a value of 80 mm/s, the maximum recommended. For temperature, a maximum value lower than the one recommended by the PLA filament manufacturer (240 °C) was selected, i.e., 225 °C and, as minimum value, 195 °C was selected that lies slightly below the minimum recommended (200 °C). Finally, values of 1 and 3 mm were selected for wall thickness, considering that wall thickness should be higher than two times the size of the nozzle extruder (0.4 mm), according to Noorani [30]. The experimental factors, along their symbols, units, and levels are listed in Table 2.

Factors and levels for experiment 1 allow generating an experimental plan to carry out experiment 1, as shown in Table 3. The experimental plan was made in a random order to guarantee that the observations or errors are independently distributed random variables [29].

The second analysis was done to specifically study the influence of wall thickness and its relation to the printing path: zig-zag, concentric, and grid. These tests were performed using the parameters used in the first stage to obtain one of the best surface roughness, so the lowest layer height was chosen (0.15 mm), but it was decided to also have a reduced printing time (estimated printing time of 47 min), so the printing speed of 80 mm/s was selected. Moreover, temperature of 225 °C was chosen. So, two factors were analyzed in this stage, using three levels for printing strategy and five for wall thickness. For printing path, concentric, zig-zag, and grid were selected. The experimental factors, along with their symbols, units, and levels are listed in Table 4.

The experiment was done using a full factorial design and the experimental tests were performed in a random order as shown in Table 5.

### 2.3. Surface Roughness Evaluation

**Figure 3:**
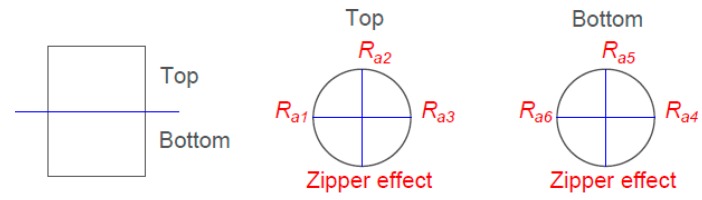
Details of the measurement procedure.

Surface roughness was evaluated in terms of the arithmetic average of the roughness profile (*Ra*). Six surface roughness measurements were taken in each sample. The samples were divided into two sections: bottom (printing start) and top (printing end) sections. In addition, three generatrices were drawn on the surface. No measurement was done in the section where the zipper effect generated by the layer change can be seen. The measurements of *Ra*_1_, *Ra*_2_, and *Ra*_3_ were taken at the top in the generatrices in a clockwise direction. The measurements of *Ra*_4_, *Ra*_5_, and *Ra*_6_ were taken at the bottom in the generatrices in a counter clockwise direction (Figure 3). Therefore, 48 and 90 measurements were obtained for experiment 1 and 2, respectively. Finally, with the six surface roughness measurements, the average roughness was calculated for each sample.

## 3. Results and Discussion

### 3.1. Surface Roughness Results

The surface roughness results obtained, their mean values and standard deviation (SD), in terms of *Ra*, are listed in Table 6 and Table 7 for experiment 1 and 2, respectively.

From the tables, it is possible to see how the values of surface roughness are high compared to other conventional manufacturing processes, such as machining. In all cases, the values are higher than 12 μm. Moreover, the results present high variability depending on the measuring point for all the tests. This variability makes it difficult to obtain conclusions on surface roughness depending on the measuring location (bottom and top). No clear trends can be found depending on the location. The standard deviation calculated for all tests show clearly this performance. It is important to see how the ranges obtained for the mean values are also high. So, for experiment 1, the values varied between 15.377 μm and 22.844 μm and, for experiment 2, they varied between 12.746 μm and 20.715 μm. Mean values for experiment 1 and experiment 2 were 19.813 and 16.460 μm, respectively. These results are used for selecting a layer height of 0.15 mm for experiment 2, expecting that the surface roughness in experiment 2 would be similar to that obtained in experiment 1.

### 3.2. Identification of Critical Factors

Statistical methods are adequate tools for identifying influential factors in datasets such as those obtained for surface roughness. Thus, Analysis of Variance (ANOVA) is performed for both experiment 1 and 2. The results are listed in Table 8 and Table 9 for experiment 1 and 2, respectively.

The normality of the residuals is checked using the Shapiro–Wilk test. Normality is verified by the calculated statistics and *p*-values: 0.86373 (W statistic) and 0.1308 (*p*-value), and 0.95369 (W statistic), and 0.5844 (*p*-value) for experiment 1 and 2, respectively. In both cases, the *p*-values are lower than the statistic, so no departure from normality was identified.

Considering that *p*-values lower than 0.05 are related to influential sources of variation, from Table 8, it is possible to recognize that layer height and wall thickness are influential factors on surface roughness. In particular, layer height has the lowest value. In addition, printing speed and temperature can be considered as nonsignificant factors for surface roughness. When analyzing the results listed in Table 9, only wall thickness is a significant source of variation, with printing path being nonsignificant.

### 3.3. Correlations between Surface Roughness and the Analyzed Factors

**Figure 4:**
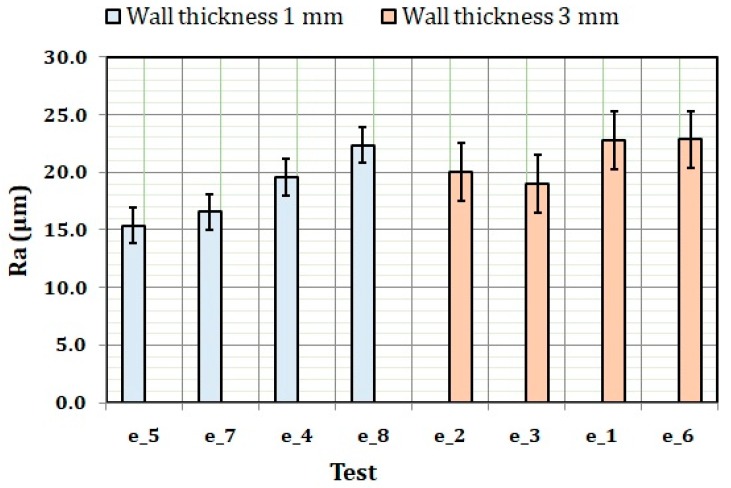
Surface roughness results of experiment 1 grouped by layer height.

In the previous section, the influential factors on surface roughness: layer height and wall thickness in experiment 1, and wall thickness in experiment 2, were identified. To evaluate the influence of these factors on surface roughness, graphical methods for identifying trends and additional statistical analysis for checking correlations were used. Based on the ANOVA results, the results of experiment 1 are plotted in Figure 4. In the figure, the tests are grouped by layer height. In the figure, it is possible to see clearly how the surface roughness obtained for the layer height of 0.15 mm is lower than the one obtained for the layer height of 0.25 mm. This result agrees well with the conventional knowledge on surface roughness obtained in additive manufacturing processes [18,31]. Moreover, it is possible to see how the lowest surface roughness was obtained for the tests that used the lowest layer height and wall thickness (e_5 and e_7).

When grouping the results by wall thickness and plotting them, it is possible to appreciate how wall thickness has a clear influence on surface roughness. Again, obviously, the results for the wall thickness of 1 mm and layer height of 0.15 mm (e_5 and e_7) are those that produced the lowest surface roughness.

**Figure 5:**
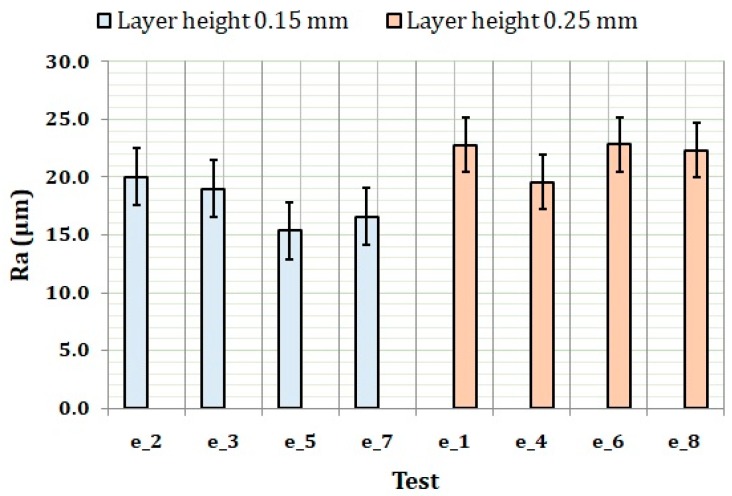
Surface roughness results of experiment 1 grouped by wall thickness.

**Figure 6:**
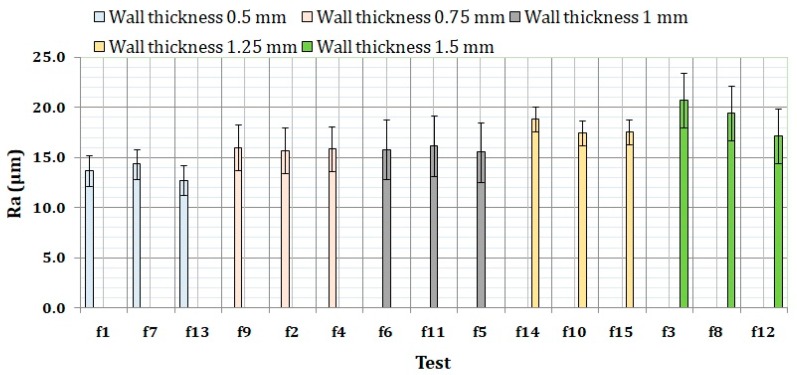
Surface roughness results of experiment 2 grouped by wall thickness.

Similar results to those obtained in Figure 5 were obtained when grouping the results of experiment 2 by wall thickness. In this case, the influence of the printing path (strategy) is negligible. In general, an increasing trend can be seen when wall thickness is increased, as seen in Figure 6. According to our best knowledge, the influence of wall thickness on surface roughness has not been previously studied in detail in the literature. In addition, a clear relation was not found between printing path strategy and wall thickness, though this relation should be studied in detail for lower values of wall thickness.

From the previous results, it is clear that both layer height and wall thickness have an important influence on surface roughness. However, the influence of the printing speed and temperature is not clear in the figures, as it was identified using the ANOVA results.

In order to confirm the influence of the different factors on surface roughness, an analysis based on the use of non-parametric tests was carried out. In this sense, Spearman’s *ρ* and Kendall’s τ correlation coefficients are calculated as done by Carou et al. [32]. These two tests are useful to identify monotonic relationships, being resistant to the effect of outliers [33]. Moreover, it is important to note that these tests do not assume a specific parametric model or specific distributions for the data [34]. The two coefficients can be calculated using Equations (1) and (2) for the Spearman’s *ρ* and Kendall’s τ, respectively [33,34].

(1) $$\rho =\frac{\sum_{i=1}^{n}\left(R_{Xi}-R_{yi}\right)-n{\left(n+1\right)}^{2}/2}{n\left(n^{2}-1\right)/2}$$

(2) $$\tau =\frac{P-M}{n\left(n-1\right)/2}$$

*n*is the number of pairs (*xi*, *yi*); *Rxi*and *Ryi* the ranks of x and y, respectively; and *P* and *M*, the numbers of pluses and minuses, respectively. R Softwarewasused for calculating the coefficients for the different factors: layer height, printing path, printing speed, temperature, and wall thickness versus surface roughness based on the results listed in Table 6 and Table 7. The results obtained are shown in Table 8. The correlation coefficients can vary from −1 (perfect negative association) to +1 (perfect positive association). When there is no correlation, the coefficient gets a value of 0 [33]. In the table, the correlation coefficients are listed along with their *p*-values.

In Table 10, similarly to the ANOVA results, it is possible to see how only layer height and wall thickness resulted as significant factors when computing the coefficients. Therefore, it is possible to state that no clear relation exists between surface roughness and printing path, printing speed, and temperature. Besides, the calculated coefficients for these relations are close to 0 (in all cases, lower than 0.3273268).

Regarding layer height and wall thickness, the coefficients have positive values. So, when increasing these two factors, higher values of surface roughness are expected. Although their values are not very close to +1, they show a monotonic correlation with values ranging from 0.47 to 0.90 for both Spearman’s *ρ* and Kendall’s τ coefficients. The results show small differences between the values obtained for these two coefficients. However, a bigger difference was found when comparing the results of experiment 1 and 2. In this case, it should be noted that only 8 experiments were carried out in experiment 1, while 15 experiments were carried out in experiment 2. So, the dataset of experiment 2 should be considered as more reliable for identifying monotonic relations. In fact, the calculated coefficients for experiment 2 show a clear correlation between surface roughness and wall thickness with *p*-value below 0.05 and values for the correlation coefficients very close to +1, while the *p*-value in the case of experiment 1 was not below 0.05.

Finally, from the graphical analysis and the statistical analysis using ANOVA and non-parametric tests, a general recommendation can be drawn. So, it is highlighted that when surface roughness is a critical requirement in additive manufacturing, particularly using FDM processes, layer height and wall thickness should be fixed at lower values. It seems clear that layer height should be as low as possible to minimize the staircase effect. However, further research should be carriedout for wall thickness to understand whether it is possible to reduce its value to a minimum or not, considering issues such as the size of the nozzle extruder and even printing path strategies that could have a negative impact when the wall thickness is too small.

## 4. Conclusions and Future Work

The present study shows an experimental investigation on surface roughness obtained in additive manufacturing processes. Fused Deposition Modeling (FDM) technology was specifically analyzed when manufacturing PLA samples. Several manufacturing parameters (layer height, printing path, printing speed, temperature, and wall thickness) were varied and the results analyzed by means of graphical and statistical analysis. The main conclusions of the investigation include the following:

- The quality of the manufactured parts depends greatly on the selection of the printing parameters. In particular, previous results that indicate that the layer height is a critical factor were validated using Analysis of Variance. But, in addition, it was found that wall thickness has an important influence on the results, which was given less attention by researchers.
- Some parameters seem to have no clear influence on the results, specifically, printing path, printing speed, and temperature. However, it should be noted that only three printing strategies were analyzed in the present study: grid, concentric, and zig-zag.
- By using Spearman’s *ρ* and Kendall’s τ correlation coefficients, the influence of layer height and wall thickness on the results was verified, especially, for experiment 2, obtaining correlation coefficients very close to +1 with *p*-values lower than 0.05.
- The effect of the layer height and wall thickness on surface roughness is to worsen the quality of the surface when one of these parameters is increased or when both are increased.
- As criteria for improving surface quality in FDM manufacturing processes, it is recommended to use reduced values of layer height, diminishing the importance of the staircase effect and also wall thickness that is generally selected based on the size of the nozzle extruder.

The results obtained in the present preliminary study will help establish new lines for future work. For instance, the influence of the material on the results should be considered; particularly the influence of the material (PLA) provided by different manufacturers should be conveniently analyzed. The use of larger datasets and higher ranges for the critical factors for verifying the results would be recommended in new experimental studies. Finally, a comprehensive analysis of the influence of wall thickness should be carried out.

## Figures and Tables

**Table 1:** Material properties.

Chemical Name	Composition	Density (g/cm^3^)	Printing Temperature (°C)	Diameter (mm)
Polylactic Acid	PLA (Polylactide Resin) 99% CAS: 9051-89-2	1.24	220 ± 20	1.75 ± 0.03

**Table 2:** Experimental factors for experiment 1.

Factor	Symbol	Units	Levels
Layer height	LH	mm	0.15, 0.25
Printing speed	PS	mm/s	40, 80
Temperature	T	°C	195, 225
Wall thickness	WT	mm	1, 3

**Table 3:** Experimental plan for experiment 1.

Test	LH (mm)	PS (mm/s)	T (°C)	WT (mm)
1	0.25	40	195	3
2	0.15	40	225	3
3	0.15	80	195	3
4	0.25	80	195	1
5	0.15	40	195	1
6	0.25	80	225	3
7	0.15	80	225	1
8	0.25	40	225	1

**Table 4:** Experimental factors for experiment 2.

Factor	Symbol	Units	Levels
Layer height	LH	mm	0.15
Printing speed	PS	mm/s	80
Temperature	T	°C	225
Printing path	PP	-	Concentric, zig-zag, grid
Wall thickness	WT	mm	0.50, 0.75, 1.00, 1.25, 1.50

**Table 5:** Experimental plan for experiment 2.

Test	LH (mm)	PS (mm/s)	T (°C)	PP	WT (mm)
1	0.15	80	225	Zig-zag	0.5
2	0.15	80	225	Grid	0.75
3	0.15	80	225	Zig-zag	1.5
4	0.15	80	225	Concentric	0.75
5	0.15	80	225	Concentric	1
6	0.15	80	225	Zig-zag	1
7	0.15	80	225	Grid	0.5
8	0.15	80	225	Concentric	1.5
9	0.15	80	225	Zig-zag	0.75
10	0.15	80	225	Concentric	1.25
11	0.15	80	225	Grid	1
12	0.15	80	225	Grid	1.5
13	0.15	80	225	Concentric	0.5
14	0.15	80	225	Zig-zag	1.25
15	0.15	80	225	Grid	1.25

**Table 6:** Experimental surface roughness results for experiment 1.

Test	*Ra*_1_ (μm)	*Ra*_2_ (μm)	*Ra*_3_ (μm)	*Ra*_4_ (μm)	*Ra_5_* (μm)	*Ra*_6_ (μm)	*Ra* (μm)	SD (μm)
1	26.045	20.202	23.188	23.284	19.558	24.358	22.773	2.474
2	20.473	20.497	20.565	17.776	18.318	22.525	20.026	1.728
3	17.937	18.46	21.145	17.205	18.182	21.051	18.997	1.680
4	19.756	17.258	21.908	18.732	19.511	20.347	19.585	1.559
5	16.066	15.252	15.338	14.842	15.239	15.524	15.377	0.405
6	25.138	24.092	25.052	19.995	20.064	22.725	22.844	2.348
7	18.252	16.929	17.705	14.073	15.709	16.697	16.561	1.499
8	23.226	23.809	23.547	21.582	19.814	22.063	22.340	1.512

**Table 7:** Experimental surface roughness results for experiment 2.

Test	*Ra*_1_ (μm)	*Ra*_2_ (μm)	*Ra*_3_ (μm)	*Ra*_4_ (μm)	*Ra*_5_ (μm)	*Ra*_6_ (μm)	*Ra* (μm)	*SD* (μm)
1	12.761	14.304	13.034	12.87	16.46	12.586	13.669	1.499
2	15.695	16.514	13.67	15.371	16.64	16.431	15.720	1.123
3	19.471	21.602	22.279	19.625	20.775	20.536	20.715	1.096
4	16.733	18.797	14.103	13.592	17.96	14.047	15.872	2.250
5	16.108	16.705	15.439	15.04	15.91	13.965	15.528	0.955
6	16.082	18.292	16.925	14.068	14.827	14.734	15.821	1.590
7	11.591	13.259	14.995	11.947	19.792	14.446	14.338	2.987
8	18.971	19.016	20.688	19.352	20.013	18.499	19.423	0.797
9	16.789	16.559	16.638	14.482	16.302	15.181	15.992	0.939
10	16.39	17.199	19.007	16.626	18.918	16.354	17.416	1.236
11	17.142	14.435	14.746	16.892	17.001	16.796	16.169	1.232
12	16.283	16.893	18.545	16.305	17.521	17.145	17.115	0.849
13	10.698	12.158	12.455	11.721	18.122	11.319	12.746	2.706
14	18.357	19.093	19.091	17.274	20.99	17.999	18.801	1.275
15	18.175	18.1	20.777	16.035	15.569	16.799	17.576	1.891

**Table 8:** Analysis of variance for experiment 1.

Source of Variation	Df	Sum sq	Mean sq	F Value	Pr (>F)
LH	1	34.366	34.366	41.3466	0.007625
PS	1	0.799	0.799	0.9619	0.399039
T	1	3.174	3.174	3.8186	0.145684
WT	1	14.518	14.518	17.4669	0.024956
Residuals	3	2.494	0.831		
Total	7	55.351			

**Table 9:** Analysis of variance for experiment 2.

Source of Variation	Df	Sum sq	Mean sq	F Value	Pr (>F)
PP	2	2.184	1.0918	1.2238	0.3438
WT	4	54.192	13.5480	15.1850	8.139 × 10^−4^
Residuals	8	7.138	0.8922		
Total	14	63.514			

**Table 10:** Correlation coefficients for experiment 1 and 2 versus the analyzed factors.

	Experiment 1		Experiment 2	
	Spearman’s *ρ*- *p*-value	Kendall’s τ- *p*-value	Spearman’s *ρ*- *p*-value	Kendall’s τ- *p*-value
Layer height	0.7637626 0.0274 *	0.6614378 0.04331 *	-	-
Printing speed	−0.1091089 0.797	−0.09449112 0.7728	-	-
Temperature	0.3273268 0.4287	0.2834734 0.3865	-	-
Wall thickness	0.5455447 0.1619	0.4724556 0.1489	0.8946933 6.729 × 10^−6^ *	0.7612299 0.0001789 *
Printing path	-	-	0.1322876 0.6384	0.1014185 0.6345

Note: * significant factor considering *p*-value < 0.05.
